# Bonobos assign meaning to food calls based on caller food preferences

**DOI:** 10.1371/journal.pone.0267574

**Published:** 2022-06-15

**Authors:** Gladez Shorland, Emilie Genty, Christof Neumann, Klaus Zuberbühler

**Affiliations:** 1 Department of Comparative Cognition, Institute of Biology, University of Neuchatel, Neuchâtel, Switzerland; 2 Cognitive Ethology Laboratory, German Primate Center, Göttingen, Germany; 3 School of Psychology and Neuroscience, University of St Andrews, St Andrews, Scotland, United Kingdom; University of Iowa, UNITED STATES

## Abstract

Human communication relies heavily on pragmatic competence. Speech utterances are often ambiguous requiring listeners to use interaction history, shared knowledge, presumed intention and other contextual variables to make inferences about a speaker’s meaning. To probe the evolutionary origins of pragmatic competence we tested whether bonobos (*Pan paniscus*) can make inferences about the type of food available from listening to other group members’ food calls. We trained two group members to either prefer blue or pink chow and demonstrated these preferences to observers. A third group member served as an untrained control. In playback experiments, we broadcast the food calls of a trained demonstrator and the untrained group member to investigate whether subjects were able to infer which coloured chow was most likely available, based on the callers’ trained food preferences or lack thereof. As predicted, when hearing the untrained group member’s calls, subjects did not exhibit a bias, whereas they responded with a significant foraging bias when hearing a trained group member’s calls. These findings suggest that bonobos may take into account the idiosyncratic food preferences of others, although subjects probably differed in what they remembered.

## Introduction

There is general agreement that the evolution of language is best studied as a collection of capacities with sometimes independent evolutionary histories, some relating to production and others to comprehension [1–3]. Since language has left no direct traces in the fossil record, a promising way to study its evolution has been to compare the cognitive and communicative capacities across primates, such as the ability to attribute meaning [e.g. 4–7], to produce signal combinations [e.g. 8–11], to communicate intentionally [e.g. 12–15] or to be socially aware [e.g. 16–18]. The logical foundation of this approach, dating back to Darwin, is that more closely related species have more similar brains than more distant ones, suggesting that their psychological capacities are also more similar [1].

Primates have limited control over their vocal output, which effectively precludes them from developing phonologies and vocal learning [19, 20]. Comprehension abilities, however, appear to be more human-like, with evidence for a general primate capacity to extract meaning from each other’s signals [20–22]. In human language, however, utterance meaning is often ambiguous and goes beyond simple signal-referent relations. To this end, humans deploy ‘pragmatics’ to infer the intended meaning of an utterance, which can deviate much from its literal meaning. This process is cognitively (and computationally) challenging and requires assessments of common ground between speakers and listeners, which can include world knowledge, cultural background, current events and socio-cognitive variables [22–24], much of which becomes accessible as the conversation unfolds. According to Grice [25], meaning in language is not just tied to lexical content but emerges as an interaction with the ongoing conversation with interlocutors taking into account a multitude of information, including context and intentions. Pragmatics, in other words, is at the interface between language and social cognition, requiring speakers to reason about other minds, specifically what information needs to be explicitly encoded, and listeners to make inferences about what the speaker has meant, two major processes likely shaped by both biological and cultural evolution [26].

Animal communication is usually seen as free from such complexities. The classic case is the alarm call system of vervet monkeys (*Cercopithecus aethiops*), with different predator alarm call types closely tied to corresponding predator encounters, allowing recipients to respond to the calls in biologically adaptive ways [4], which also suggests that alarm calls are meaningful to them [2]. This model of relatively rigid call-referent relations has often been used to interpret findings in primate communication, including calls given to food [9, 27–31], predators [e.g. 7, 32] or during within-group aggression [e.g. 33, 34]. Meaning, in this sense, is situated in the ‘functionally referential’ connection between external events and the signals they trigger.

More recently, it has been argued that this model of meaning is insufficient to cover the full range of phenomena in animal communication [35] and it certainly is insufficient to explain human communication. While animal communication always takes place within a context that is shared by signallers and receivers [36], it is not clear how this impacts on whether or how animals encode and extract meaning [20, 37]. In one chimpanzee study, subjects were able to infer the nature of out-of-sight social interactions from listening to call exchanges [34]. When subjects heard sequences of victim and aggressor screams, they looked longer at incongruent sequences (i.e., low-ranking individual’s aggressor screams and high-ranking individual’s victim screams, hence violating existing dominance relations) than congruent ones (low-ranking victim screams and high-ranking aggressor screams, hence in accordance with the hierarchy), suggesting that they made inferences about third-party interactions they could not see. In other studies with monkeys, subjects responded differently to their own and other species’ alarm calls depending on the additional context provided to them [38, 39].

Inferential (or causal) reasoning has been researched in various domains of animal cognition [40], but not usually in communication research. Current theory posits that only humans are able to represent events in terms of an underlying causal structure, which enables them to make inferences about absent entities beyond simple associatively learned stimulus response relations [41]. Human children are thought to possess a ‘causal map’ of the world, which consists of learned representations of the relations among events in the form of a Bayesian network [42]. Whether or to what degree primates and other animals have inferential capacities of this kind is under-researched and controversial. In one study, for example, chimpanzees were unable to use auditory cues to predict the location of a reward dropped through an opaque conductor [43], whereas in another study subjects from four great ape species correctly identified the location of hidden food with the help of both visual and auditory cues [42]. Similarly, across experimental conditions, great apes were able to select boards that were likely to cover food due to their inclined orientation, either due to abstract inferences or simply having learned that inclined orientations predict food [44].

Our goal was to address great ape inferential capacities in the domain of communication, a topic with special relevance for the evolution of language, especially the origins of pragmatics. We use the term ‘pragmatic inference’ in a broad sense, by referring to an ability to incorporate knowledge about a signaller in order to enrich the interpretation of the signal. Importantly, we do not refer to a key aspect of linguistic pragmatics, i.e., the ability to infer communicative or informative intention [45]. Instead, we sought to investigate more broadly whether bonobos were generally able to enrich the meaning of calls by adding knowledge related to call producers, a likely precursor of the human ability to form common ground and other higher forms of social cognition. Common ground refers to the shared mental state that two interlocutors, as signaller and recipient, establish and maintain during a conversation. Here, we only looked at the recipient side, not at the interactive process underlying the formation of common ground.

We chose food calls because this class of vocalisations has already been investigated in previous studies. When interacting with food, bonobos produce sequences of acoustically distinct vocalisations, depending on the perceived quality of food [9, 27]. In particular, sequences to highly preferred foods typically, but not exclusively, contain barks and peeps, whereas sequences to less preferred foods typically, but not exclusively, contain peep-yelps and yelps [27]. The different call types are graded and sequence composition is highly variable [9, 27]. Despite this high level of plasticity in sequence composition, recipients are able to make inferences about the type of food available [9]. In one analysis it was found that duplications of call types within a sequence appeared to be especially influential on how recipients assign meaning [46], but this needs to be tested with targeted experiments. Previous research has also shown that bonobo food calls, like many other primate vocalisations, are individually distinct [9, 27, 47], with experimental evidence that individuals recognise each other by their call sequences over long periods of time [48].

In our study, we first trained two group members to have opposing food preferences for two artificially coloured food items, pink or blue chow, such that KEL preferred blue and DW preferred pink, while a third group member, LNG, served as an untrained control. In previous research we have established that bonobos can develop socially learned preferences for familiar foods with changed visual appearance from artificial colouration, even if they otherwise taste and look the same [49]. After having observed KEL and DW executing their individual preferences for blue and pink chow, respectively, we then asked whether other group members took these individual demonstrator preferences into account when making their own foraging decisions. To test this, we designed a playback study, using recordings of KEL, DW and LNG’s food calls. We predicted that, if subjects’ foraging decisions varied depending on who was the demonstrator, then this would be relevant evidence for evolutionary theories of pragmatic inference. Understanding that other individuals have food preferences that deviate from one’s own is akin to understanding something about their mental states and allowing predictions about their intentions. Importantly, in this sense the current study goes significantly beyond what has been demonstrated, i.e., that chimpanzees and bonobos can make inferences about the presence of generally preferred and generally not-preferred foods [9, 50].

## Method

### Study site and subjects

Research was carried out between February 2015 and October 2016 with a group of bonobos at ‘La Vallée des Singes’ primate park, Romagne, France. The group of 20 individuals (9 males and 11 females, 0–46 years, see S1 Table) lived in an indoor enclosure (400m^2^) composed of 10 interconnected cages with access to two outdoor wooded islands (11,500m^2^). Three individuals (a mother with her adult and subadult sons) were separated from the group in December 2015 in preparation for departure to a different zoo and did not participate in the study.

### Experimental design

The experiment sought to simulate a situation in which a subject could make inferences about the type and location of food, based on who provided the information. To this end, and as explained earlier, we trained two individuals (KEL, DW) to prefer either blue or pink chow (S1.1 Text in S1 Text), while a third individual (LNG) served as a control with no trained preference. To establish these food preferences, we used monkey chow that was either left to its natural taste or artificially made bitter with Bitrex^®^ while changing its visual appearance from natural brown to artificially coloured pink or blue with household food colouring (see S1.2 Text in S1 Text, S1 Fig). Methods further described in Shorland *et al*. [49].

Once the two opposing colour preferences were established in KEL and DW, subjects observed KEL and DW during feeding events. As it should be, KEL consistently preferred blue chow (while avoiding pink chow) and DW consistently preferred pink chow (while avoiding blue chow). Since chow was a highly valued food, both demonstrators regularly produced food calls during training in response to their respectively coloured chow, as all individuals did when consuming unaltered chow during regular feeding events.

As a second key experience, subjects learned that, on a given day, only one type of coloured chow (blue or pink) was available and only in its corresponding location. Since blue chow was always given in one trough and pink always in the other, subjects learned that if the blue chow trough was baited, then the pink chow trough, by definition, was empty. Hence, each chow colour was only available alone, at one specific location and for an entire day. Although this would have been desirable, it was technically not possible to individually check that each subject understood this contingency and it is possible that some subjects simply did not pay attention to the pattern or were unable to remember it.

After these two training experiences, we carried out the playback experiment, which consisted of subjects hearing recorded food calls from a trained demonstrator or from the untrained control individual. We tested whether subjects took into account whose calls they heard, before visiting the food locations (see S2 Fig for further details). We predicted that exploration behaviour, i.e., peering and probing (see definitions below) following playback of the untrained LNG’s calls should be arbitrary, since LNG had no colour preference (hence making it impossible to predict which coloured chow was available), whereas exploration behaviour following playback of the two trained demonstrators should be side-biased, either toward the blue trough (KEL) or the pink trough (DW), respectively.

### Phase 1: Preference demonstration phase

**Fig 1:**
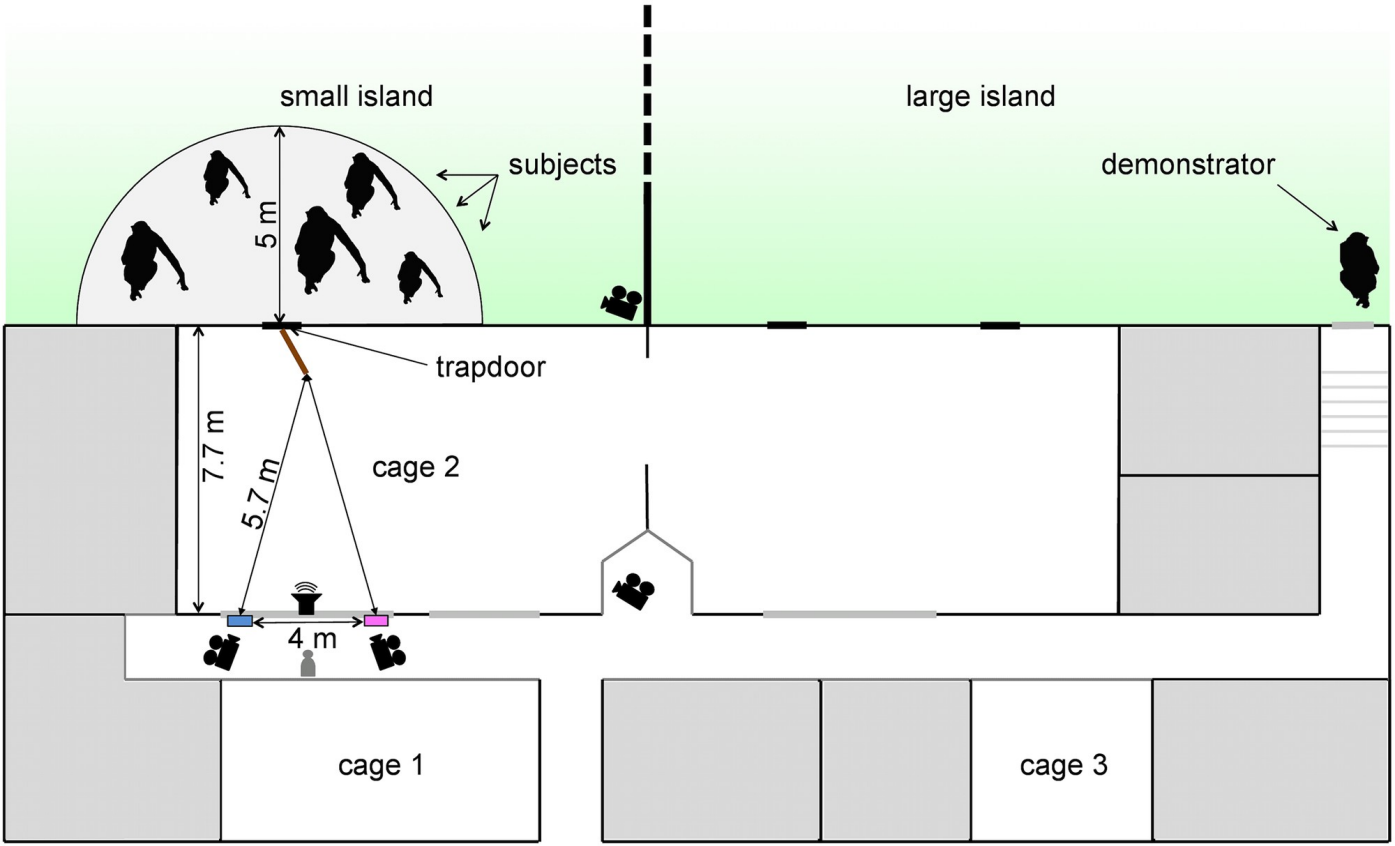
Layout of indoor and outdoor enclosures for the three experimental phases. *Phase 1 –observational learning*: demonstrators (KEL, DW) were held individually in cage 1 (demonstration room) and given a choice of pink and blue chow. Subjects were able to observe this process from across the corridor in cage 2 (observation room). *Phase 2 –foraging training*: blue or pink chow was provided to the subjects in one of two adjacent food troughs, 4 metres apart and equidistant from the point of entry (trapdoor). *Phase 3 –playback experiment*: a loudspeaker was placed at midpoint between the two food troughs. The demonstrator whose calls were to be played back was lured away to the large island so as to be out of earshot, while subjects were required to be on the small island within 5 metres of the trapdoor. During each trial, the experimenter (GS) positioned herself at midpoint between the two troughs to document the subjects’ foraging behaviour.

In a previous study, we established that bonobos could learn individually distinct, arbitrary food preferences of other group members through rapid social learning and by mere observation [49]. In both the previous and the current study, KEL and DW served as demonstrators who preferred either blue (KEL) or pink (DW) chow. During preference demonstrations, artificially dyed blue or pink chow was offered to the demonstrators manually and in full view of the subjects (Fig 1). When demonstrating their preferences before others, both individuals showed very clear preferences for their assigned colours (first choices: KEL–blue 99.6%, DW–pink 100%), providing subjects with an unambiguous learning opportunity.

During the experimental period 24 preference demonstrations were carried out by each demonstrator. An initial demonstration phase, consisting of three demonstration days for each demonstrator, took place in April 2015, a reminder period involving four demonstration days for each demonstrator ensued in June 2015. Following this, refresher preference demonstrations took place approximately every 3–4 weeks until the experiment was over. Preference demonstrations consisted of the experimenter (GS) placing two chow pellets of each colour (i.e., four total) on two identical white plastic trays (20 x 20 cm), placed side by side against the bars of the cage, allowing the demonstrator to select them using either his fingers or lips (S3 Fig). The position (left or right) of blue or pink food items was counter-balanced and randomly determined, with the restriction that a given colour could not be presented on the same side for more than three consecutive trials (for supplementary information see S1.3 Text in S1 Text).

Both the demonstrators and the observers were filmed to document demonstrator choices and observer attention (PANASONIC HC-V100 and PANASONIC HC-V727 full HD cameras). Subjects observing from outside the camera range were included with help from a trained animal keeper. Observer attention was defined as head and eyes oriented towards the demonstrator whilst a choice was made and coded as attending (1) or not attending (0) [49].

### Phase 2: Foraging training

Two white plastic feeding troughs (51x10x8cm) were fixed to the bars of cage 2 at two points, 4 metres apart and equidistant from the entrance from the small island (Fig 1). Blue chow was always supplied in the left trough, pink chow always in the right one (this rule was randomly determined). The content of the troughs was visible to subjects only when standing right next to and peering into them (S4 Fig). On a given day, only one trough was baited with coloured chow, which was supplied throughout the day whenever an individual approached the trough (S2 Table).

To avoid conflict at the troughs, we opted against ad libitum food supply throughout the day. Instead, we provided the chow in an intermitted and controlled way, baiting the predetermined trough only when an individual approached and only when two or three individuals (not including dependant infants) were present in the surrounding area. In particular, if several high-ranking individuals were present, baiting was suspended to avoid causing escalated conflicts. After each successful baiting, the experimenter then returned to the mid-point between the two troughs to document all subsequent behaviours before returning to the trough for the next round of baiting. This controlled way of delivering coloured chow also allowed us to make sure that the two demonstrators did not become ‘untrained’ by accidentally consuming the other coloured chow. For example, if KEL (blue preference) approached the pink trough we did not give him any pink chow, even though all other group members were able to obtain it. Hence, although the demonstrators were permitted to feed at the troughs, this was only possible for their preferred food colour. We implemented this extra condition in order to increase the plausibility of the subsequent playback experiment.

Foraging training with the group started in March 2015. In a first block of 30 days, group members could learn that pink or blue chow was regularly available at specific locations (i.e., feeding troughs) in the experimental enclosure (Fig 1; cage 2) on 20 baiting days (10 per trough) interspersed with 10 resting days when the experimenter (GS) was present but no trough was baited. Baiting condition (pink, blue, nothing) was predetermined randomly, although the same condition could not occur for more than two consecutive days. Initially, we planned to carry out the subsequent playback experiments in May 2015, but for technical reasons this had to be postponed. We therefore restarted foraging training in June 2015 as a refresher for a period of six days (4 baiting days, i.e., 2 per trough, interspersed with 2 resting days). From July 2015 onwards and throughout the entire playback phase regular colour-trough reminders were carried out (approx. 4–5 per month) according to a predetermined random order (no more than two consecutive reminders for a given trough).

Subjects’ choices were documented using a PANASONIC HC-V100 full HD camera. A second PANASONIC HC-V727 full HD camera was set up in front of the non-baited trough to create identical conditions.

### Phase 3: Playback experiment

We obtained recordings of food calls given to chow, a food highly valued by all individuals, from the three call providers (KEL: blue preference; DW: pink preference; LNG: no colour preference). Recordings were made opportunistically between March 2015 and May 2016 while individuals were feeding on natural chow during normal scatter feeds. For KEL we carried out 10 recording sessions from which 10 call sequences were obtained, for DW nine recording sessions with 10 sequences retained and for LNG 12 recording sessions with six sequences retained (S5 Fig, S1 Audio, for supplementary information see S1.4 Text in S1 Text). As explained earlier, bonobos produce highly variable sequences to foods, with high proportions of barks and peeps to highly valued foods and high proportions of peep-yelps and yelps to less valued foods [9, 46].

Call sequences were recorded at a close distance using a MARANTZ PMD660 solid-state recorder and a SENNHEISER MKH416T directional microphone for subsequent editing on an APPLE MacBook Pro using Raven Pro 1.4 (WAV files; 44.1kHz sampling rate, 16-bit accuracy). We only included call sequences that were free from excessive background noise and overlap with other calls. Playback stimuli were made from these recordings and always consisted of series of four calls, edited from the first four calls of natural sequences, with inter-call intervals edited to shorter than one second if necessary (see S5 Fig), resulting in a total duration of about 4s for each playback sequence (10.6084/m9.figshare.12665300).

The experiment was based on the generally accepted notion that, like other primates, bonobos recognise each other by their calls, something that is empirically established for many types of primate vocalisations [51–67].

A precondition for successful individual recognition is that there are consistent acoustic differences between individuals for a given call type. For bonobo vocalisations, evidence for reliable individual differences in call structure exists for copulation calls [27], food calls [9] and attention-getting peep calls [47]. Importantly, in a playback experiment Keenan *et al*. [48] used food calls to demonstrate that bonobos (including four individuals from the current study group) were able to recognise individuals from their voices alone, even after prolonged periods of separation of up to 5 years, in line with the currently accepted theory that voice recognition is a general feature of primate cognition [68, 69].

All playback sequences were broadcast from a speaker positioned between the two troughs in the experimental enclosure (Fig 1; cage 2) and only when all individuals were outside. Importantly, neither trough was baited during playback trials to ensure that subjects could use the playback stimuli as the only basis for decisions. Before each trial, the respective call provider was lured away with food onto the large island (Fig 1), out of earshot to avoid hearing his or her own calls [70].

However, despite considerable efforts, we did not manage to separate DW (the pink demonstrator) from the group in this way, which unfortunately ruled him out as call provider in the playback experiment, a major weakness of this study. Although it would have been possible to train another group member to replace DW as a pink demonstrator, we decided against this solution, for two reasons. First, both KEL and LNG (but not DW) naturally spent much of their time separated from the group, which made it relatively easy to lure them away prior to a playback trial. All remaining group members were far more gregarious, so there was no obvious candidate who could have been lured away easily. Second, even if such an individual existed, it would have created an imbalance in the number of preference demonstrations between KEL (blue) and the new demonstrator (pink). Overall, given the time and effort necessary to implant a food preference, to then demonstrate this preference to others) and the lack of another suitable demonstrator, we opted for a simple comparison of performance after hearing the vocalisations of KEL (trained) and LNG (untrained), hoping that future studies will eventually address this shortfall.

Playback conditions thus consisted of subjects hearing a series of food calls from KEL (blue preference) and LNG (no preference) from within the experimental enclosure. To this end, we lured the call provider away to the large island and then ensured that (a) no individual was in the experimental enclosure (Fig 1; cage 2) and (b) at least one individual was within a 5m radius in the outside area directly adjacent to the trap door leading to cage 2 (Fig 1), i.e., within earshot of the playback stimulus. To augment the attractiveness of this area we provided small quantities of grain at irregular intervals. The area in front of the trap door was surveyed with an AQUILA VIZION Smartvizion FIX’ HD 720P IP camera, positioned on the roof of the building, above cage 2 (Fig 1). The camera was linked via Ethernet to a LINKSYS WRT54G Wireless-G router from which the image was transmitted to an iPad via Wi-Fi (S6 Fig for example of image), allowing the experimenter inside to decide when the trial could be initiated.

Once all conditions were met, a playback trial was initiated, which consisted of broadcasting a sequence of four food calls by KEL or LNG. Stimuli were played via iTunes on an APPLE MacBook Pro using a BOSE SoundLink Mini Bluetooth Speaker (approx. frequency range: 70Hz-8kHz; power: 8W). In pilot trials, we adjusted volume settings to obtain naturally sounding amplitudes, as judged by a keeper positioned in the outside area near the trap door, the future location of subjects.

#### Coding

As mentioned, no food was provided during the playback trials, to the effect that subjects always encountered empty troughs after responding to the playback. During the entire playback phase (which lasted 398 days) we thus added 60 refresher days (30 per feeding trough) to ensure subjects retained the previously established routine of the experimenter regularly baiting the feeding troughs.

An individual became a subject for a given trial as soon as it entered cage 2 through the trap door and approached one of the troughs to either show exploration or expectation. Following each playback trial, subject reactions were recorded over a period of one hour. This allowed us to observe and record the behaviour of a maximum number of individuals upon entering cage 2, despite considerable variation in latency. We opted for this analysis window because low-ranking individuals were often prevented from entering cage 2, apparently waiting for the more competitive individuals to enter and leave the cage. Moreover, although the stimulus was clearly heard outside, we often observed subjects to continue with ongoing activities, which often delayed their entering the building until having finished their previous activity.

Our prediction was that, if subjects could (a) relate the calls to KEL or LNG and (b) knew that only KEL had a food preference (as witnessed during training), then they should anticipate blue chow after hearing KEL’s calls and anticipate either blue or pink chow after hearing LNG’s food calls.

### Dependent variables

The most obvious measure would have been to compare ‘approach’ frequencies to each feeding trough across playback conditions. However, we found that individuals sometimes entered cage 2 and approached a trough but then showed no intentions to forage, suggesting that they were not interested in food (data for ‘approach’ frequencies is presented in S3 Table). We therefore decided to only consider trials in which subjects exhibited interest in food, by ‘approaching to interact’ with the experimenter or by ‘approaching to explore’ the trough content.

With ‘approaching to interact’ we were interested in whether subjects showed signs of expectation towards the experimenter to refill the trough that, presumably, had previously been emptied by the call provider. We therefore measured the proportion of time spent looking at the experimenter while located within a designated zone (S7 Fig; S1 Video). With ‘approaching to explore’, we were interested in actual foraging behaviour that we classified as either (a) peering into the trough and (b) probing the trough with fingers (S2 Video). Predictions were that the exploratory behaviour (peering & probing) following playback of the untrained LNG’s calls should not be side-biased (since she had no food preference, making it impossible to predict which side to find food). In contrast, exploratory behaviour following playback of the two trained demonstrators should be side-biased, either toward the blue trough (KEL) or the pink trough (DW), respectively.

We used three cameras, two positioned in the corridor in front of each trough and a third one with an overall view of cage 2 (see Fig 1 for camera disposition; two PANASONIC HC-V727 full HD cameras and one PANASONIC HC-V100 full HD camera; for supplementary information on criterion for subject participation see S1.5 Text in S1 Text). Data coding was carried out by GS on an APPLE MacBook Pro using Squared 5 software MPEG Streamclip 1.9.2^©^.

### Statistical analyses

We ran two analyses, one for ‘expectation’ and one for ‘exploration’. First, we modelled ‘expectation’ as the response variable with a generalised linear mixed model (GLMM) with binomial error structure. The response variable was the proportion of time spent looking at the experimenter out of the total time a subject spent in that colour zone. The interaction between test condition (call provider with preference versus call provider without) and trough (blue versus pink) was the main predictor variable. We also included a binary control predictor, which indicated whether the subject had attended (or not) to the last demonstration event prior to a given playback trial (‘demonstration exposure’). Subject identity and playback trial were included as random intercepts. We tested the full model against the null model (which included the demonstration exposure main effect, subject identity and playback trial as random intercepts) with a likelihood ratio test (LRT [71]).

Second, we modelled ‘exploration’ using a GLMM with binomial error structure, i.e., whether or not the subject explored the correct trough. As before, the interaction between test condition and trough was the main predictor variable. Demonstration exposure was included as a control predictor, subject identity and playback trial were included as random intercepts, and included total time spent in the colour zone as offset term. We tested the full model against a null model (which included the demonstration exposure main effect, and subject identity and playback trial as random intercepts) with a likelihood ratio test (LRT [71]).

Finally, we considered the individual mean frequencies of the two exploratory behaviours, peer and probe, separately. The sample size for probing was too low for any statistical analyses. For peering, however, we were able to run a non-parametric Wilcoxon signed rank test to compare each individual’s Δ (mean frequency of peering at the pink trough minus mean frequency of peering at the blue trough = delta) between the test (KEL) and control (LNG) conditions.

Statistical analyses were carried out using R v. 3.4.3 and lme4 v. 1.1–15. In the supplemental documents, we provide the script as a tool kit (S1 and S2 Files) to further explore the raw data.

### Compliance with ethical standards

The study was in line with recommendations in the ARRIVE guidelines and the EAZA and AFdPZ code of ethics, authorised and given ethical approval by the “La Vallée des Singes” scientific coordinator and zoological director. Although KEL and LNG were separated from the group during the playback trials, this was on a voluntary basis in order to get privileged feeding opportunities and for short periods of time (<30 min), with no signs of stress. When social conditions within the group were tense (e.g., during aggression), testing was postponed.

## Results

As the group was large with individuals free to move around, meeting all required playback conditions was an extremely challenging task, resulting in a total of N = 12 playback trials (N = 6 in the test condition, i.e., KEL’s food calls, indicating blue preference; and N = 6 in the control condition, i.e., LNG’s food calls, indicating no preference) over a period of 13 months (Sept 2015 –Oct 2016). We managed to test N = 10 subjects (3 males; 7 females, age range: 5–46 years; S1 Table) with N = 9 completing at least one trial in both conditions (test: N = 10, control: N = 9). This was because LNG as the call provider could not participate as a subject in the control condition. Two infants (MO, KLS) also took part but were excluded as they were still dependent on their mothers. The average number of subjects per trial was 4.5 (median = 4.5, range = 1–8, N = 12 trials).

For ‘expectation’, we found that subjects generally spent little time looking towards the experimenter, regardless of experimental condition. The full model was not significantly different from the null model (Generalized linear mixed model, likelihood ratio test (LRT): Chi^2^ = 4.20, d.f. = 3, P = 0.2409, S8 Fig, S4 Table), suggesting that (contrary to predictions) the playback stimuli did not trigger obvious and systematic expectation towards the experimenter.

**Fig 2:**
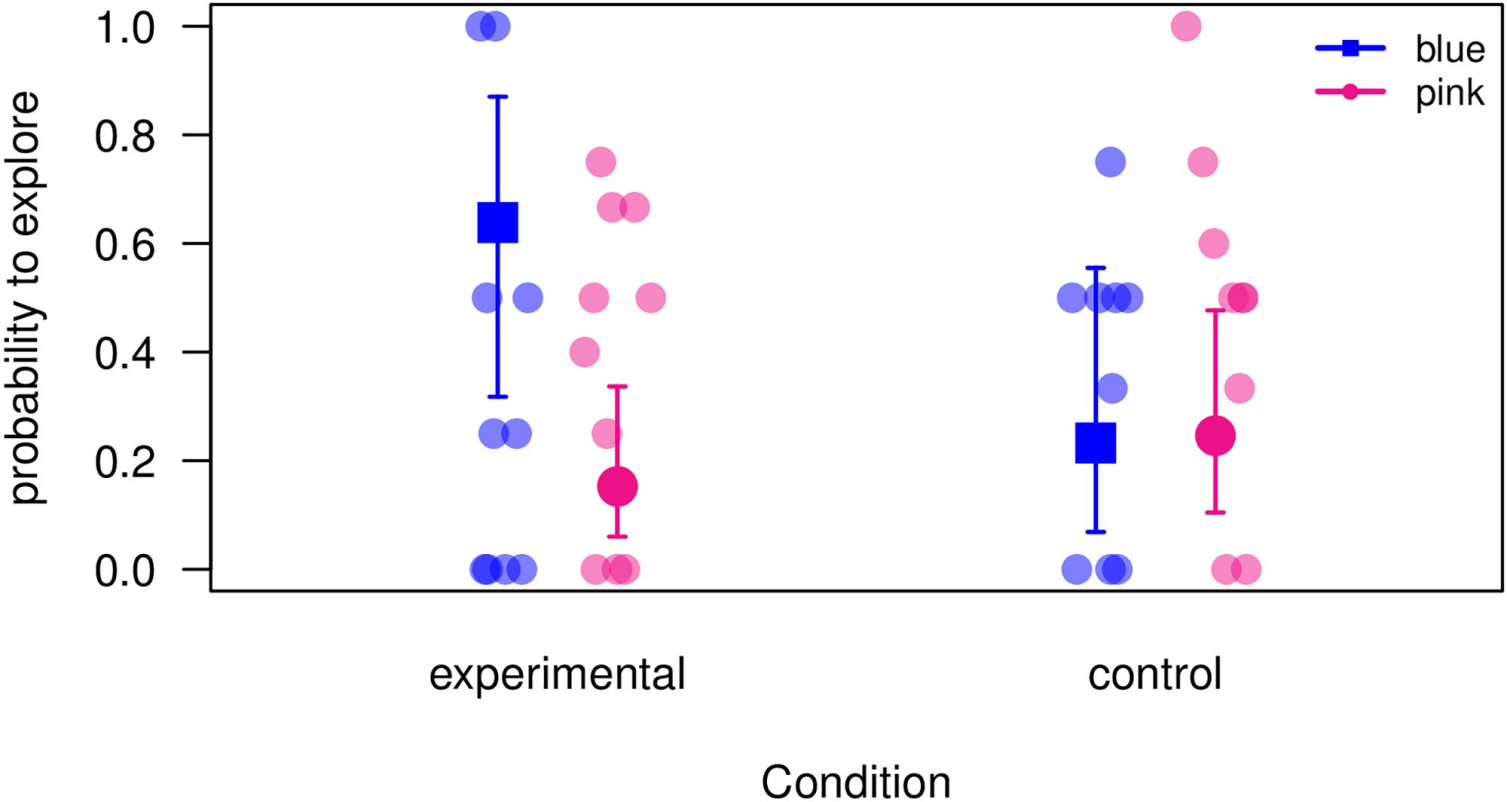
Exploration. Probability of subjects exploring the blue or pink food troughs in test and control conditions. Circles represent the average probability to explore for each subject (10 subjects participated in the 6 test trials and 9 subjects participated in the 6 control trials). Squares with bars represent model estimates with 95% confidence intervals.

For ‘exploration’ (i.e., peering and probing analysed together), results were in the predicted direction when comparing the full and null models (LRT: Chi^2^ = 6.97, d.f. = 3, P = 0.0728, Fig 2, S5 Table). In the test condition (KEL calls, indicating blue preference), individuals were overall more likely to explore the blue (correct) trough compared to the pink (incorrect) trough, but this was due to three individuals (DV, LNG, NK), who performed in line with predictions. It should be noted that the significance of the statistical model was largely due to one individual, NK (see S1 and S2 Files: removing NK has the strongest effect on the model). The remaining individuals were either more likely to explore the pink trough, or showed the same number of responses to both troughs.

In the control condition (LNG calls, indicating no food preference), individuals were overall equally likely to explore both the blue and the pink trough. The majority of subjects (6 of 9) were in line with our predictions by showing similar interest in both troughs.

**Fig 3:**
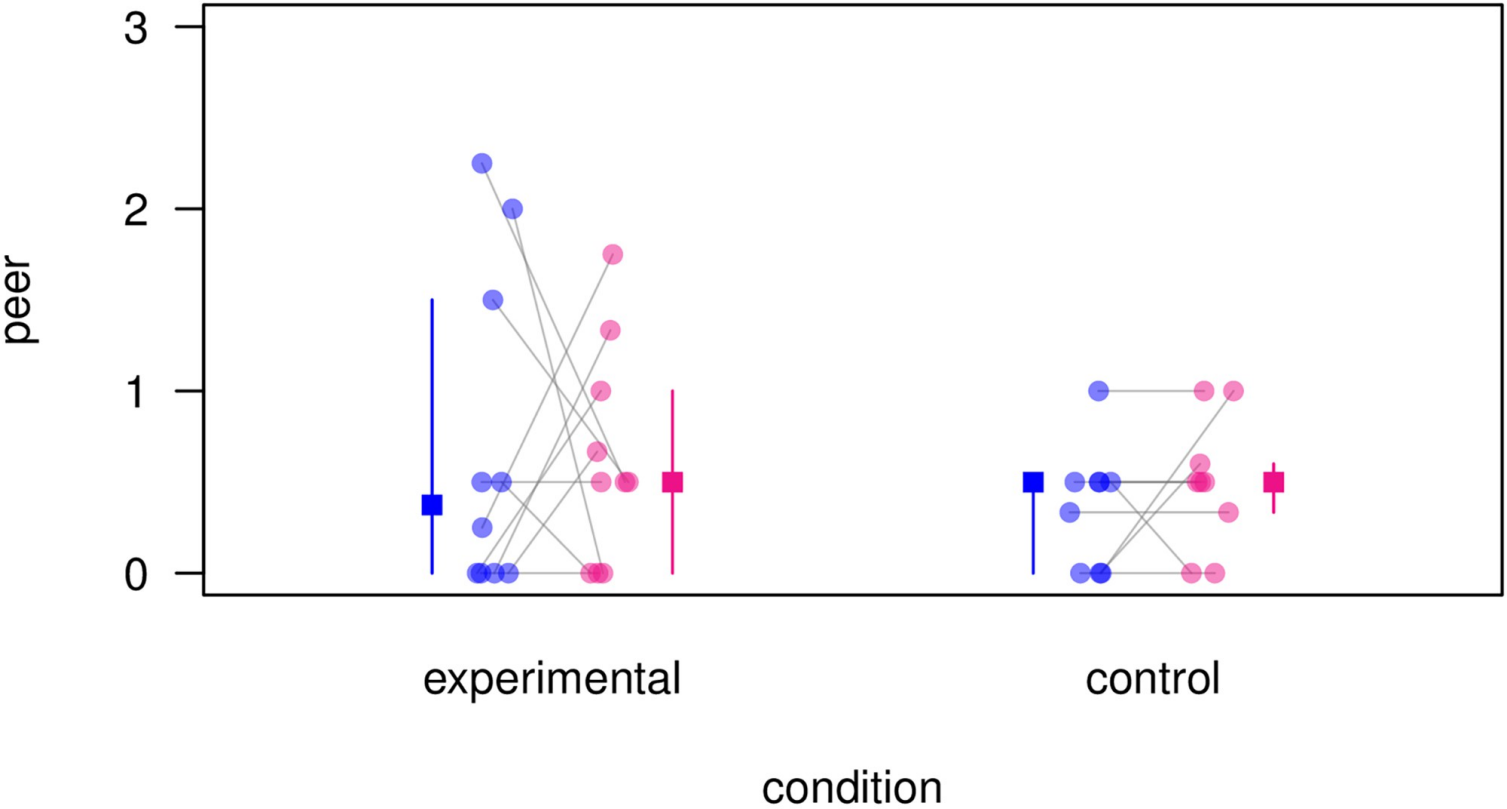
Peering frequency. Mean frequency of peering across individuals at the blue and pink food troughs in test and control trials. Circles represent mean frequencies of peering across individuals (10 subjects participated in the 6 test trials and 9 subjects participated in the 6 control trials). Squares with bars represent medians with 25% and 75% quartiles. Subjects in the test condition show higher bias for one of the two troughs than subjects in the control condition.

We then analysed the components of exploration separately and found that ‘probing’ was generally rare, with only 5 of 10 subjects exhibiting the behaviour (see S1 and S2 Files), which prevented us from carrying out further analyses. For ‘peering’, however, we found a significant (undirected) trough bias in the test condition relative to the control condition (Fig 3): Seven of nine subjects showed a trough bias (peering more in the blue *or* pink trough) after hearing KEL’s calls, while only three of nine subjects showed a trough bias after hearing LNG’s calls (KEL: blue: N = 3, pink: N = 4; LNG: blue: N = 1, pink: N = 2, see S9 Fig; Wilcoxon signed rank test, N = 6 (2 ties), V = 21, P = 0.036, two-tailed).

We encourage the reader to further explore our raw data (S1 Data) and analyses with the online interactive tool provided in the supplemental information (S1 and S2 Files).

## Discussion

The aim of this study was to investigate whether bonobos were able to draw inferences from knowledge about other individuals’ food preferences previously acquired by observational learning. The design was based on the species’ demonstrated capacity for individual vocal recognition [48] and their documented propensity to vocalise when encountering highly valued food, such as chow [9, 27]. We predicted that, when hearing the calls of a demonstrator with a specific and previously demonstrated food preference, subjects should explore the location where the demonstrator’s preferred food was usually available (see Fig 1: left trough for blue chow; right trough for pink chow). We also predicted that, when hearing the calls of another individual, with no known food preference (LNG), subjects should not show such a bias.

To test our hypothesis, we provided subjects with social learning opportunities. First, subjects observed that two of their group members (KEL, DW) had opposing preferences regarding coloured chow while a third individual (LNG) served as a no preference control. Subjects also learned that blue and pink chow were available in fixed locations, but never simultaneously. Once this was established, we simulated food was available in inside by playing back food calls by KEL (blue preference) or LNG (no preference).

We monitored subjects upon entry and did not find a clear preference for approaching one trough over the other, neither in the experimental nor the control condition. Upon arrival at a trough, we measured foraging behaviour, exploration and expectation, and found an effect for exploration in the predicted direction (Fig 2), although there was considerable inter-individual variation. When analysing peering and probing separately, we found that for most subjects peering was significantly biased towards one trough (blue or pink) when responding to KEL’s calls, but not when responding to LNG’s calls (Fig 3). Interestingly, the highest peering rates were in the experimental condition, i.e., when responding to blue-trained KEL’s food calls, whereas peering rates to untrained LNG’s food calls were substantially lower. This may be due to the fact that subjects could make a prediction with KEL’s calls, and may have approached the troughs in a more determined and anticipatory way, whereas this was not possible in response to LNG’s calls.

From these data we concluded that at least some subjects recognised KEL as someone with a chow colour preference and LNG as someone without such a preference. Three subjects (DV, LNG, NK) behaved as if they not only knew that KEL had a colour preference but also that it was a preference for blue chow. Clearly, the task in this experiment was complex, requiring subjects to form various associations about the food preferences of others and knowledge about where and when to find the corresponding foods. First, subjects had to associate the identity of a demonstrator to his preferred chow colour, then to associate the two chow colours to two trough locations, before finally being able to make an inference that a demonstrator’s food calls were indicative of the availability of a specific food at a specific location. Although subjects were provided with extensive opportunities to learn these associations, our data suggest that not all animals were able to form them or to recall them during the playback experiments. For example, rather than remembering KEL as someone who preferred *blue* chow, some subjects may have formed a less sophisticated representation, i.e., that KEL had *some* colour preference, but not for which one (see results). It is also possible that some individuals simply did not remember where the blue and pink foods were provisioned. Although they were given reminders (4–5 per month, food colour and location) it is not certain that they were able to track these locations over time. To address this hypothesis, it would have been necessary to carry out another test, but this would have further increased the complexity of the experimental design. In any case, individual differences in cognitive capacities are well documented in great apes [72] and this may have led to some individuals not learning the colour/location association. Naturally, there may have been other reasons to explain our finding, such as differences in attention during the demonstration phase or carryover effects of the refresher baiting routine. Nevertheless, the fact that KEL’s food calls caused a foraging bias, whereas LNG’s food calls did not, is in line with the hypothesis that bonobos are able to draw basic inferences about others’ food preferences, although there appear to be individual differences in the complexity of the underlying mental representations.

How do these results compare to other research on primate call comprehension? As mentioned, field experiments with Old World monkeys have demonstrated that non-human primates can take context into account when responding to each other’s calls, across call types [38, 39, 73]. However, in all previous studies the context was always the same for the caller and the recipients, in contrast to the current study. This is also true for the previous playback study on bonobo food calls [9], where there was no disagreement between call providers and their audiences regarding the referents, since all subjects preferred kiwi over apples.

## Conclusions

While we acknowledge that the task was complex, our data suggest that at least three individuals performed in line with the hypothesis that bonobos are capable of some pragmatic inference, or at least an essential component of this capacity. Other subjects also behaved as if they recognised the call provider as someone with a known food preference (KEL), which differed from their responses to another call provider with no known food preference (LNG). We take these results to suggest that—at a very basic level—bonobos take others’ individual preferences into account, even if they differ from their own, although our study does not reveal much about the content of this knowledge.

If these results can be confirmed by future work, they have clear implications for current theories of language evolution and the debate about the origins of Gricean communication. Data also bear relevance to the question of whether pragmatic competence was a precursor to language or co-evolved with it. The coevolution hypothesis rests on the assumption that language and social cognition, particularly mindreading, emerged together as suggested by simulation experiments [74]. The precursor hypothesis is that only humans possess socio-cognitive abilities required for Gricean communication and that they operated as precursors for language evolution and ontogenetic pre-requisites for language development [75, 76]. A more moderate view is that the socio-cognitive abilities necessary for language evolved gradually, reaching beyond the last common ancestor. Human pragmatic abilities, in this view, are mere improvements of more basic socio-cognitive abilities, which now operate in conjunction with significantly increased processing power to enable language [77]. On the production side, other-mind awareness, partially demonstrated by this study, is ostensive communication (pointing, showing), which is difficult to demonstrate in great apes [78]. Our data are in line with the notion that Gricean pragmatics evolved gradually, starting with an early ability to make inferences about how others operate, an ability that must have emerged before chimpanzees, bonobos and humans split into separate lines.

## Supporting information

S1 Fig Coloured chow. Pink and blue monkey chow for use in foraging training and preference demonstrations.
(PDF)

S2 Fig Three phases of the experiment. 1) Observational learning: Subjects were provided with the opportunity to learn by observation the association between a demonstrator (KEL, DW) and its preferred food colour (*A-B*); 2) Foraging training: Subjects were provided with the opportunity to learn by individual experience and observation the association between food colour and a specific food trough (*B-C*); 3) Playback experiment: Tested whether subjects were able to associate the identity of a call provider (KEL, LNG) to a food trough (A-C). Phases 1 & 2 were carried out simultaneously and continued in between playback trials of phase 3.
(PDF)

S3 Fig Food presentation. Presentation of pink and blue chow to KEL during a preference demonstration in full view of the subjects, using 20 x 20cm white plastic trays.
(PDF)

S4 Fig Baited feeding trough. Pink, baited, feeding trough (dimensions 51x10x8 cm).
(PDF)

S5 Fig Stimulus spectrogram. Example of a stimulus from KEL used for the playback experiment. Clicks used to mark calls from focal individual have been removed and natural call intervals preserved.
(PDF)

S6 Fig View of outdoor enclosure. Example of the view from the rooftop IP camera surveying the 5-metre radius and surrounding areas in front of the trapdoor on the small island.
(PDF)

S7 Fig Experimental set-up. Experimental set-up (wide-angle view) showing the designated blue and pink zones and respective food troughs where subject behaviour was coded. The loudspeaker is located at the centre point.
(PDF)

S8 Fig Expectation. Proportion of time spent looking at the experimenter (i.e., expectation) at the blue or pink feeding troughs. Circles represent the average proportion of time spent looking at the experimenter (expectation) for each subject (10 subjects participated in the six test trials and 9 subjects participated in the six control trials). Squares with bars represent model estimates with 95% confidence intervals.
(PDF)

S9 Fig Mean peering frequency delta. Peer—Mean frequency delta (i.e., absolute difference: mean frequency of peering at pink minus mean frequency of peering at blue = Δ) between the two food locations in test (full bars) and control (empty bars) conditions. Colours indicate the direction of the trough bias. (10 subjects participated in the six test trials and 9 subjects participated in the six control trials). Two of the ten subjects were excluded from this analysis: UK, as she expressed peering behaviour not once and LNG, as she participated only in the test condition.
(PDF)

S1 Table Study group composition. Group composition at La Vallée des Singes, Romagne, France, and role of different group members in the present study.
(PDF)

S2 Table Foraging training for subjects. (PDF)

S3 Table Subject approach frequency. Frequency at which each feeding trough was first approached by each subject in the experimental and control conditions.
(PDF)

S4 Table Result of the GLMM testing for differences in expectation behaviour. (PDF)

S5 Table Result of the GLMM testing for differences in exploration behaviour. (PDF)

S1 Text Supplementary information. (PDF)

S1 Audio Stimulus example. (WAV)

S1 Video Expectation. Subject looking at the experimenter while standing in the designated area close to a feeding trough.
(MP4)

S2 Video Exploration. Subject probing the feeding trough with fingers while standing in the designated area close to a feeding trough.
(MP4)

S1 Data Raw data. (CSV)

S1 File Interactive results manual. Manual for the shorlandetal package for use with R.
(PDF)

S2 File Supporting file for interactive results: Shorlandetal package. (GZ)
