# Supplementary figures and images for: Bonobos assign meaning to food calls based on caller food preferences
Source: PLoS One. 2022 Jun 15;17(6):e0267574. doi: 10.1371/journal.pone.0267574 (PMC9200338; doi:10.1371/journal.pone.0267574)

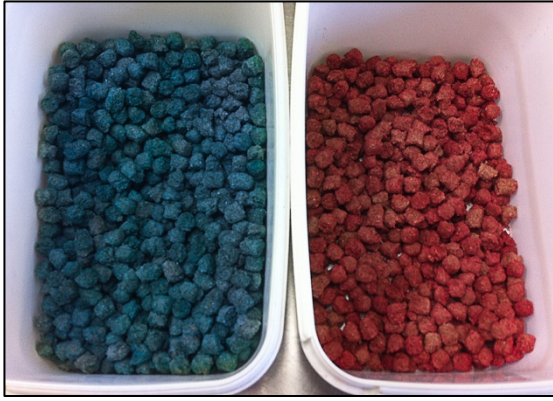

**Figure S1.** Pink and blue monkey chow for use in foraging training and preference demonstrations.

Supplement: S1 Fig — Pink and blue monkey chow for use in foraging training and preference demonstrations. (PDF) [file pone.0267574.s001.pdf]

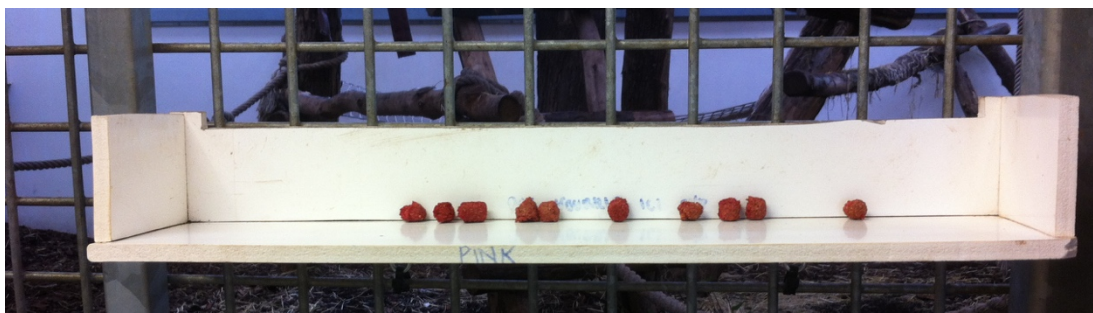

**Figure S4.** Pink, baited, feeding trough (dimensions 51x10x8 cm)

Supplement: S4 Fig — Pink, baited, feeding trough (dimensions 51x10x8 cm). (PDF) [file pone.0267574.s004.pdf]
