# Supplementary material for: Bonobos assign meaning to food calls based on caller food preferences
Source: PLoS One. 2022 Jun 15;17(6):e0267574. doi: 10.1371/journal.pone.0267574 (PMC9200338; doi:10.1371/journal.pone.0267574)
Supplement: S2 Fig — 1) Observational learning: Subjects were provided with the opportunity to learn by observation the association between a demonstrator (KEL, DW) and its preferred food colour (A-B); 2) Foraging training: Subjects were provided with the opportunity to learn by individual experience and observation the association between food colour and a specific food trough (B-C); 3) Playback experiment: Tested whether subjects were able to associate the identity of a call provider (KEL, LNG) to a food trough (A-C). Phases 1 & 2 were carried out simultaneously and continued in between playback trials of phase 3. (PDF) [file pone.0267574.s002.pdf]

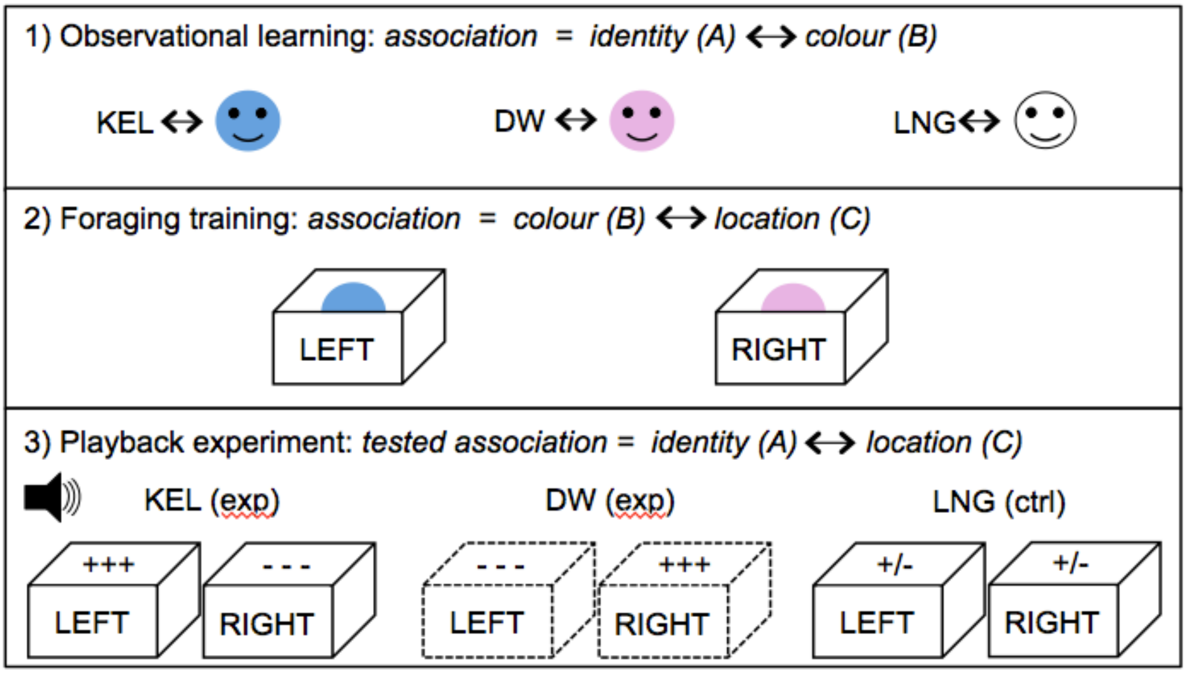


**Figure S2.** Three phases of the experiment: 1) Observational learning: Subjects were provided with the opportunity to learn by observation the association between a demonstrator (KEL, DW) and its preferred food colour (*A*-*B*); 2) Foraging training: Subjects were provided with the opportunity to learn by individual experience and observation the association between food colour and a specific food trough (*B*-*C*); 3) Playback experiment: Tested whether subjects were able to associate the identity of a call provider (KEL, LNG) to a food trough (A-C). Phases 1 & 2 were carried out simultaneously and continued in between playback trials of phase 3.
