# Supplementary material for: Bonobos assign meaning to food calls based on caller food preferences
Source: PLoS One. 2022 Jun 15;17(6):e0267574. doi: 10.1371/journal.pone.0267574 (PMC9200338; doi:10.1371/journal.pone.0267574)
Supplement: S3 Fig — Presentation of pink and blue chow to KEL during a preference demonstration in full view of the subjects, using 20 x 20cm white plastic trays. (PDF) [file pone.0267574.s003.pdf]

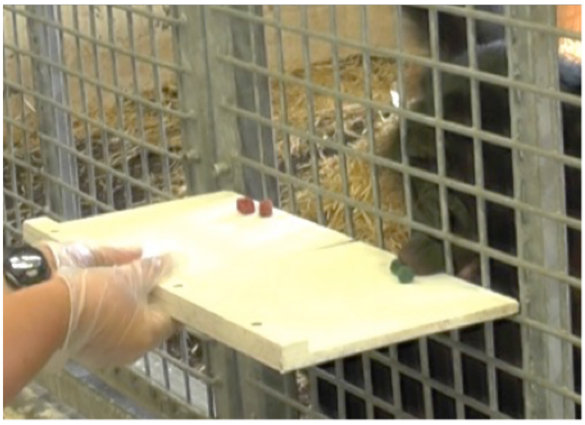

Figure S3. Presentation of pink and blue chow to KEL during a preference demonstration in full view of the subjects, using 20 x 20cm white plastic trays.
