# Supplementary material for: Bonobos assign meaning to food calls based on caller food preferences
Source: PLoS One. 2022 Jun 15;17(6):e0267574. doi: 10.1371/journal.pone.0267574 (PMC9200338; doi:10.1371/journal.pone.0267574)
Supplement: S5 Fig — Example of a stimulus from KEL used for the playback experiment. Clicks used to mark calls from focal individual have been removed and natural call intervals preserved. (PDF) [file pone.0267574.s005.pdf]

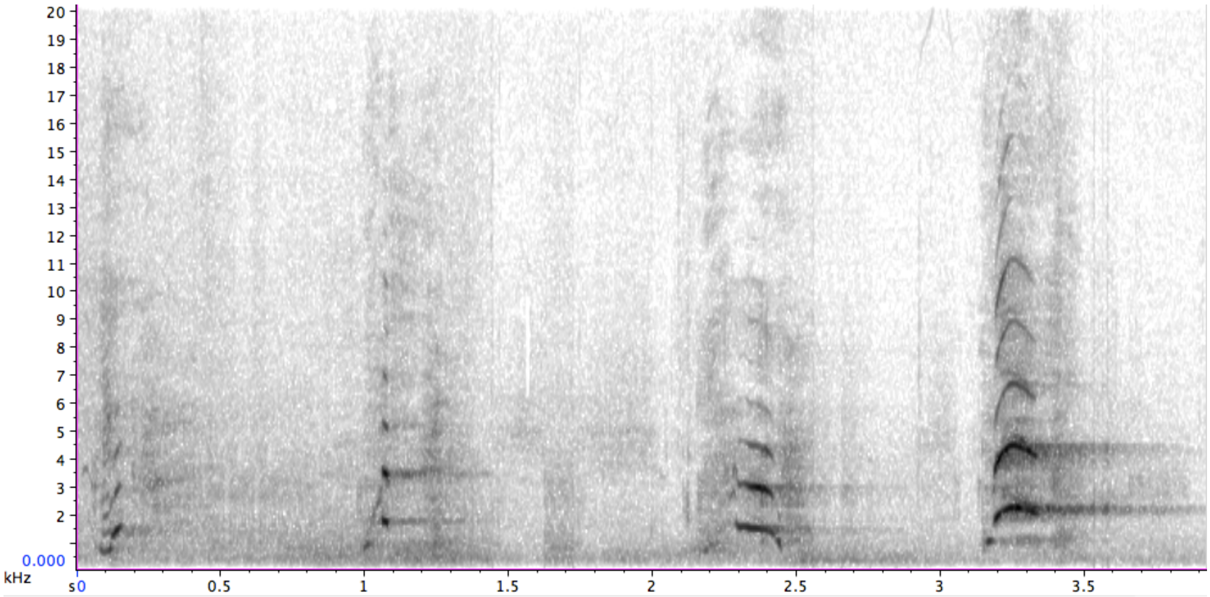

Figure S5. Example of a stimulus from KEL used for the playback experiment. Clicks used to mark calls from focal individual have been removed and natural call intervals preserved.
