# Supplementary material for: Bonobos assign meaning to food calls based on caller food preferences
Source: PLoS One. 2022 Jun 15;17(6):e0267574. doi: 10.1371/journal.pone.0267574 (PMC9200338; doi:10.1371/journal.pone.0267574)
Supplement: S6 Fig — Example of the view from the rooftop IP camera surveying the 5-metre radius and surrounding areas in front of the trapdoor on the small island. (PDF) [file pone.0267574.s006.pdf]

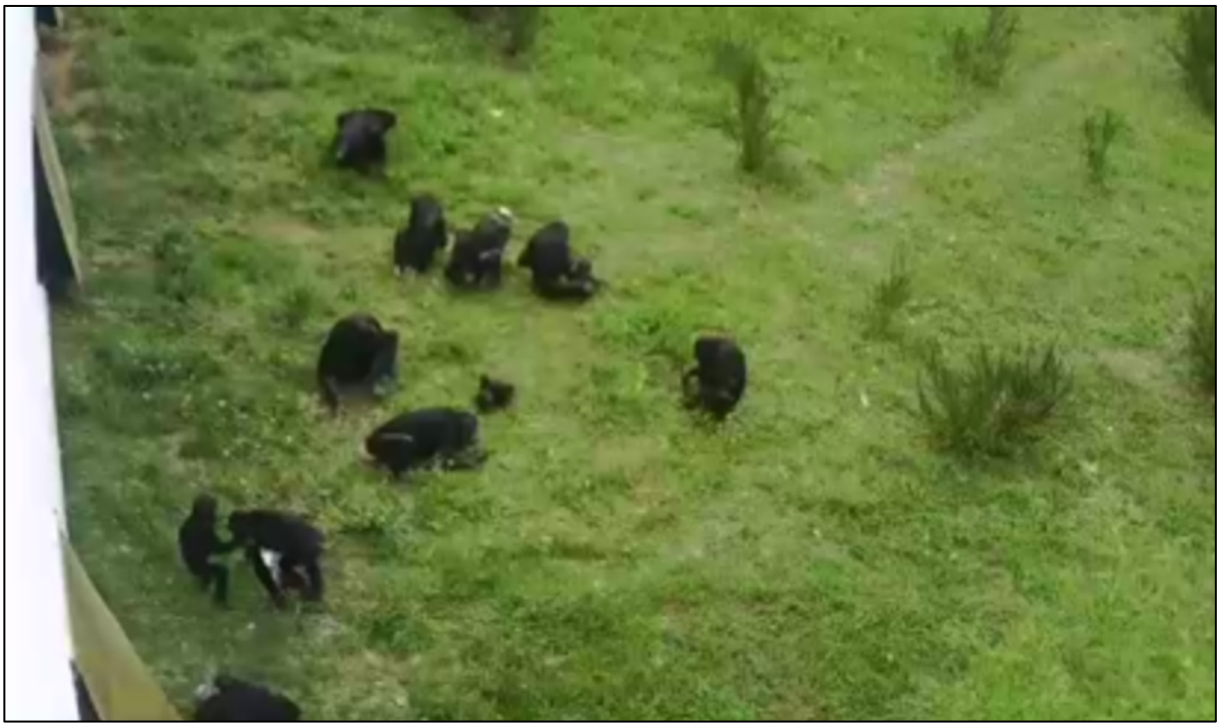

Figure S6. Example of the view from the rooftop IP camera surveying the 5-metre radius and surrounding areas in front of the trapdoor on the small island
