# Supplementary material for: Bonobos assign meaning to food calls based on caller food preferences
Source: PLoS One. 2022 Jun 15;17(6):e0267574. doi: 10.1371/journal.pone.0267574 (PMC9200338; doi:10.1371/journal.pone.0267574)
Supplement: S7 Fig — Experimental set-up (wide-angle view) showing the designated blue and pink zones and respective food troughs where subject behaviour was coded. The loudspeaker is located at the centre point. (PDF) [file pone.0267574.s007.pdf]

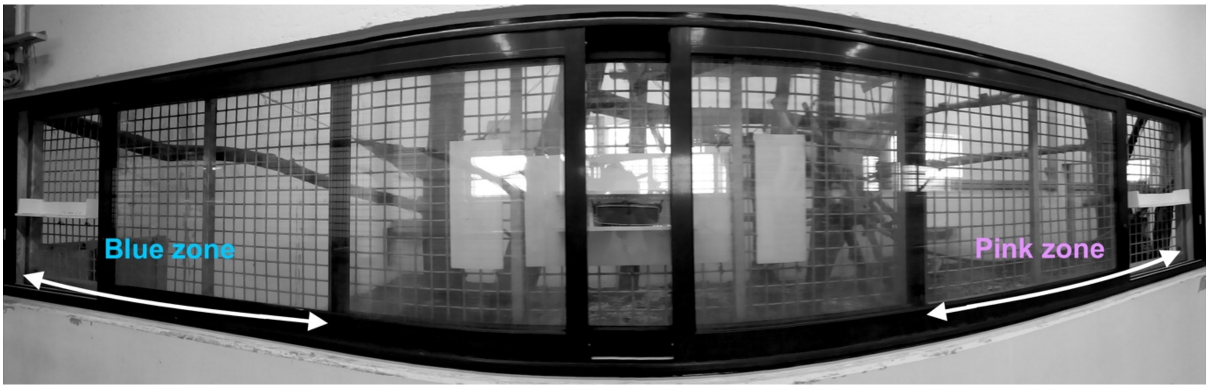

Figure S7. Experimental set-up (wide-angle view) showing the designated blue and pink zones and respective food troughs where subject behaviour was coded. The loudspeaker is located at the centre point.
