# Supplementary material for: Bonobos assign meaning to food calls based on caller food preferences
Source: PLoS One. 2022 Jun 15;17(6):e0267574. doi: 10.1371/journal.pone.0267574 (PMC9200338; doi:10.1371/journal.pone.0267574)
Supplement: S8 Fig — Proportion of time spent looking at the experimenter (i.e., expectation) at the blue or pink feeding troughs. Circles represent the average proportion of time spent looking at the experimenter (expectation) for each subject (10 subjects participated in the six test trials and 9 subjects participated in the six control trials). Squares with bars represent model estimates with 95% confidence intervals. (PDF) [file pone.0267574.s008.pdf]

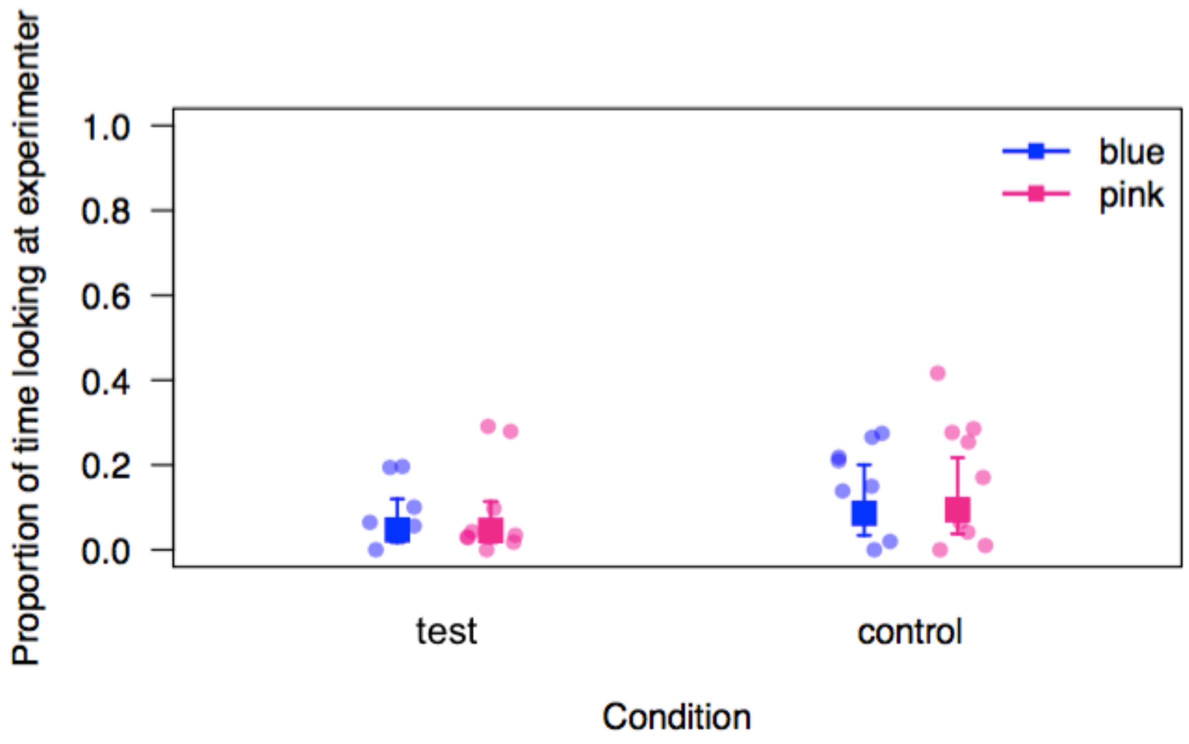

Figure S8. Proportion of time spent looking at the experimenter (i.e. expectation) at the blue or pink feeding troughs. Circles represent the average proportion of time spent looking at the experimenter (expectation) for each subject (10 subjects participated in the six test trials and 9 subjects participated in the six control trials). Squares with bars represent model estimates with 95% confidence intervals.
