# Supplementary material for: Bonobos assign meaning to food calls based on caller food preferences
Source: PLoS One. 2022 Jun 15;17(6):e0267574. doi: 10.1371/journal.pone.0267574 (PMC9200338; doi:10.1371/journal.pone.0267574)
Supplement: S9 Fig — Peer—Mean frequency delta (i.e., absolute difference: mean frequency of peering at pink minus mean frequency of peering at blue = Δ) between the two food locations in test (full bars) and control (empty bars) conditions. Colours indicate the direction of the trough bias. (10 subjects participated in the six test trials and 9 subjects participated in the six control trials). Two of the ten subjects were excluded from this analysis: UK, as she expressed peering behaviour not once and LNG, as she participated only in the test condition. (PDF) [file pone.0267574.s009.pdf]

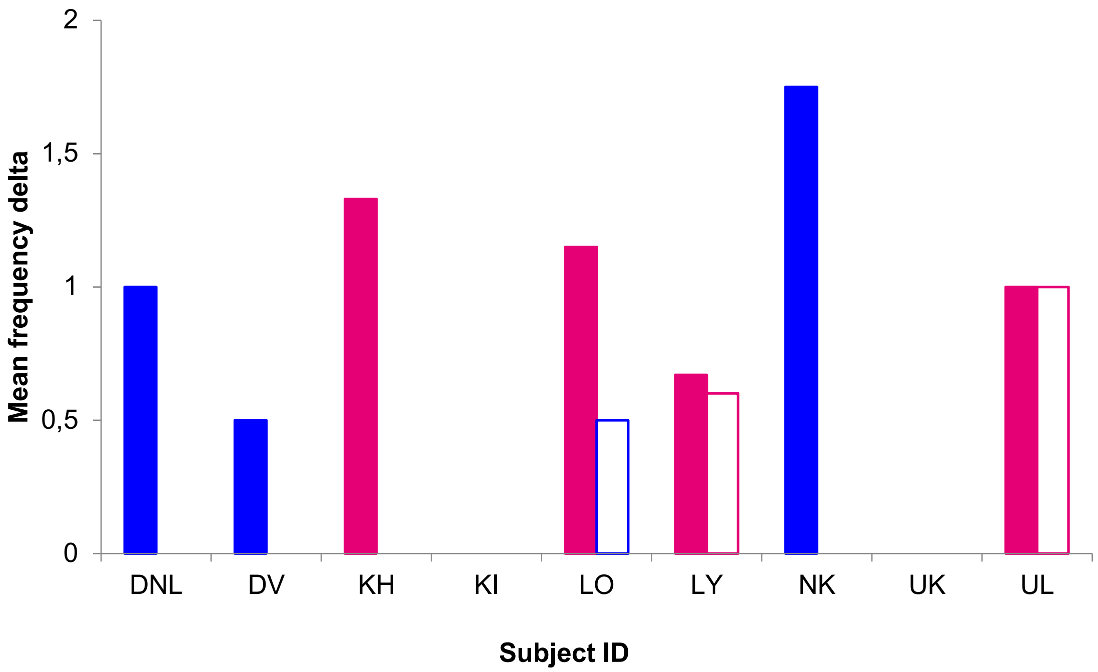

Figure S9. Peer - Mean frequency delta (i.e., absolute difference : mean frequency of peering at pink minus mean frequency of peering at blue = Δ) between the two food locations in test (full bars) and control (empty bars) conditions. Colours indicate the direction of the trough bias. (10 subjects participated in the six test trials and 9 subjects participated in the six control trials). Two of the ten subjects were excluded from this analysis: UK, as she expressed peering behaviour not once and LNG, as she participated only in the test condition.
