# Supplementary material for: Bonobos assign meaning to food calls based on caller food preferences
Source: PLoS One. 2022 Jun 15;17(6):e0267574. doi: 10.1371/journal.pone.0267574 (PMC9200338; doi:10.1371/journal.pone.0267574)
Supplement: S1 Table — Group composition at La Vallée des Singes, Romagne, France, and role of different group members in the present study. (PDF) [file pone.0267574.s010.pdf]

Table S1. Group composition at La Vallée des Singes, Romagne, France, and role of different group members in the present study

Name	Code	Sex	Birth year	Age-class	Role
Diwani	DW	M	1996	Adult	Demonstrator
Kelele	KEL	M	2004	Subadult	Demonstrator
Daniela	DNL	F	1968	Adult	Test subject
Lisala	LSL	F	1980	Adult	*
Ukela	UK	F	1985	Adult	Test subject
Bondo	BO	M	1991	Adult	*
Kirembo	KI	M	1992	Adult	Test subject
Ulindi	UL	F	1993	Adult	Test subject
David	DV	M	2001	Adult	Test subject
Khaya	KH	F	2001	Adult	Test subject
Lingala	LNG	F	2003	Subadult	Test subject & Control
Lucy	LY	F	2003	Subadult	Test subject
Luebo	LUE	M	2006	Subadult	*
Nakala	NK	F	2007	Subadult	Test subject
Loto	LO	M	2009	Juvenile	Test subject
Moko	MO	M	2012	Infant	
Khalessi	KLS	F	2012	Infant	
Yuli	YU	F	2014	Infant	
Swahili	SWH	F	2014	Infant	
Lokoro	LKR	M	2016	Infant	

Note: Demonstrators (N=2), control (N=1), test subjects (N=10). * Individuals separated from main group (N=3). Age-class, as defined by Kano (1984), at the start of the study.
