# Supplementary material for: Bonobos assign meaning to food calls based on caller food preferences
Source: PLoS One. 2022 Jun 15;17(6):e0267574. doi: 10.1371/journal.pone.0267574 (PMC9200338; doi:10.1371/journal.pone.0267574)
Supplement: S2 Table — (PDF) [file pone.0267574.s011.pdf]

Foraging training for subjects

Troughs were not baited every day. Baiting of the troughs was carried out by the experimenter (GS), when individuals were present. After baiting, the experimenter returned to the mid-point between the two troughs (see fig. 1). Attention to surrounding individuals and the hierarchy was necessary when baiting in order to avoid competition and potential conflict (e.g. if too many high ranking individuals were present at the trough, baiting was suspended until fewer individuals were present).

The first of these training periods in March and April was comprised of 20 baiting days (10 per feeding trough) interspersed with days when no trough was baited. Selection for the baited trough (pink, blue or none) was predetermined and pseudorandomised, the same trough was baited for no more than 2 consecutive days. A further 4 days training were carried out in June and yet another 4 in July bringing the total number of foraging training days to 14 per feeding trough. From the end of July onwards, throughout the playback experiment, an additional 60 refresher days (30 per feeding trough) were put in place in order to maintain the habituation of the group to the experimenter baiting the feeding troughs.

March - July 2015
Total PINK = 14, BLUE = 14

July 2015 - October 2016
Total PINK = 30, BLUE = 30

	Date	Baited trough		Date	Baited trough
1	05/03/2015	PINK	1	22/07/2015	PINK
2	06/03/2015	BLUE	2	23/07/2015	BLUE
3	11/03/2015	BLUE	3	06/08/2015	BLUE
4	12/03/2015	PINK	4	07/08/2015	PINK
5	17/03/2015	BLUE	5	12/08/2015	BLUE
6	18/03/2015	PINK	6	19/08/2015	PINK
7	20/03/2015	PINK	7	26/08/2015	BLUE
8	21/03/2015	BLUE	8	02/09/2015	PINK
9	24/03/2015	BLUE	9	08/09/2015	PINK
10	26/03/2015	PINK	10	15/09/2015	BLUE
11	27/03/2015	PINK	11	23/09/2015	PINK
12	28/03/2015	BLUE	12	29/09/2015	BLUE
13	30/03/2015	PINK	13	11/10/2015	BLUE
14	01/04/2015	BLUE	14	11/11/2015	PINK
15	04/04/2015	PINK	15	14/11/2015	BLUE
16	05/04/2015	BLUE	16	20/11/2015	PINK
17	06/04/2015	PINK	17	23/11/2015	PINK
18	07/04/2015	BLUE	18	25/11/2015	BLUE
19	08/04/2015	BLUE	19	03/12/2015	BLUE
20	09/04/2015	PINK	20	07/12/2015	PINK
21	05/06/2015	PINK	21	08/12/2015	BLUE
22	08/06/2015	BLUE	22	10/12/2015	PINK

23	11/06/2015	PINK
24	13/06/2015	BLUE
25	01/07/2015	PINK
26	02/07/2015	BLUE
27	16/07/2015	BLUE
28	17/07/2015	PINK

23	13/01/2016	BLUE
24	14/01/2016	PINK
25	18/01/2016	BLUE
26	22/01/2016	PINK
27	26/01/2016	BLUE
28	27/01/2016	BLUE
29	28/01/2016	PINK
30	25/04/2016	PINK
31	28/04/2016	PINK
32	29/04/2016	BLUE
33	01/05/2016	BLUE
34	04/05/2016	PINK
35	06/05/2016	BLUE
36	07/05/2016	PINK
37	10/05/2016	PINK
38	14/05/2016	BLUE
39	19/05/2016	PINK
40	24/05/2016	PINK
41	03/06/2016	BLUE
42	06/06/2016	PINK
43	14/06/2016	BLUE
44	22/06/2016	BLUE
45	27/06/2016	PINK
46	08/07/2016	BLUE
47	25/07/2016	PINK
48	27/07/2016	BLUE
49	03/08/2016	PINK
50	04/08/2016	BLUE
51	09/08/2016	BLUE
52	11/08/2016	PINK
53	29/08/2016	BLUE
54	30/08/2016	PINK
55	01/09/2016	PINK
56	02/09/2016	BLUE
57	20/09/2016	PINK
58	21/09/2016	BLUE
59	27/09/2016	BLUE
60	03/10/2016	PINK
