# Supplementary material for: Bonobos assign meaning to food calls based on caller food preferences
Source: PLoS One. 2022 Jun 15;17(6):e0267574. doi: 10.1371/journal.pone.0267574 (PMC9200338; doi:10.1371/journal.pone.0267574)
Supplement: S3 Table — Frequency at which each feeding trough was first approached by each subject in the experimental and control conditions. (PDF) [file pone.0267574.s012.pdf]

Table S3. Frequency at which each feeding trough was first approached by each subject in the experimental and control conditions.

Name	Experimental		Control	
	Pink	Blue	Pink	Blue
DNL	1	1	4	0
UK	1	0	1	0
KI	3	1	2	2
UL	5	0	3	0
DV	1	1	1	1
KH	3	0	3	1
LNG	0	1	NA	NA
LY	3	0	5	0
NK	0	4	3	0
LO	4	0	2	0
