# Supplementary material for: Bonobos assign meaning to food calls based on caller food preferences
Source: PLoS One. 2022 Jun 15;17(6):e0267574. doi: 10.1371/journal.pone.0267574 (PMC9200338; doi:10.1371/journal.pone.0267574)
Supplement: S4 Table — (PDF) [file pone.0267574.s013.pdf]

**Table S4.** Result of the GLMM testing for differences in expectation behaviour

| | Estimate | Standard error | *Z* |
|---|---|---|---|
| Intercept | -2.89 | 0.51 | -5.69 |
| Demonstration exposure (no versus yes) | 1.04 | 0.15 | 6.81 |
| Condition (control versus test) | -0.65 | 0.47 | -1.39 |
| Location (blue versus pink) | 0.10 | 0.07 | 1.37 |
| Interaction (condition*location) | -0.13 | 0.21 | -0.64 |

Note: Terms in parentheses indicate the two levels for each factor.
