# Supplementary material for: Bonobos assign meaning to food calls based on caller food preferences
Source: PLoS One. 2022 Jun 15;17(6):e0267574. doi: 10.1371/journal.pone.0267574 (PMC9200338; doi:10.1371/journal.pone.0267574)
Supplement: S5 Table — (PDF) [file pone.0267574.s014.pdf]

Table S5. Result of the GLMM testing for differences in exploration behaviour.

	Estimate	Standard error	*Z*
Intercept	-4.62	0.79	-5.82
Demonstration exposure (no versus yes)	0.94	0.62	1.51
Condition (control versus test)	1.78	0.93	1.91
Location (blue versus pink)	0.13	0.83	0.15
Interaction: condition*location	-2.36	1.14	-2.07

Note: Terms in parentheses indicate the two levels for each factor.
