# Supplementary material for: Bonobos assign meaning to food calls based on caller food preferences
Source: PLoS One. 2022 Jun 15;17(6):e0267574. doi: 10.1371/journal.pone.0267574 (PMC9200338; doi:10.1371/journal.pone.0267574)
Supplement: S1 Text — (PDF) [file pone.0267574.s015.pdf]

Text S1.1 *Demonstrator preference reinforcement*

We trained one demonstrator (KEL, a low-ranking adult male) to prefer artificially coloured blue chow over alternatively coloured pink chow. We trained another demonstrator (DW, a medium-ranking adult male) with the opposite preference (pink over blue chow). This training, along with the subsequent preference demonstrations, required frequent isolation from the group, which both KEL and DW, probably due to their lower social standing, endured without apparent signs of stress and without causing obvious tension in the group. Both KEL and DW had been trained for these specific food colour preferences in a prior experiment (Shorland *et al.*, 2019). Both demonstrators successfully completed their training in which they were each isolated on two consecutive days and presented with the choice of blue or pink chow. Finally, we identified a control individual (LNG, a low-ranking subadult female), who did not undergo any preference training.

In a previous experiment (Shorland *et al.*, 2019), both demonstrators, DW and KEL, had gained experience with artificially coloured foods and their associated tastes. Nonetheless, a learning period was necessary in order to introduce the new coloured food type (chow) and reinforce their manipulated colour preference. On two consecutive days, the demonstrators were isolated one after another in a cage with **no vis-à-vis** (see cage 3, fig. 1) and were presented with the two coloured food items ten consecutive times. Food presentation lasted 10 seconds after a first choice had been made. DW was presented with natural tasting pink and bitter blue chow (see text S1.2 for details about food preparation) and KEL was presented with natural tasting blue and bitter pink chow. Both individuals used their previous knowledge regarding the colour-taste associations and immediately chose the palatable colour first. This process was carried out twice, once for an initial learning phase and again as a reminder previous to the Preference demonstrations in full view of the subjects. Demonstrator preference reinforcement was

recorded using a PANASONIC HC-V727 full HD camera equipped with a SENNHEISER MKE 400 external microphone.

Text S1.2 *Food preparation and presentation*

Monkey chow (SDS® Old World Monkey Chunks, henceforth, chow) was used throughout the experiment. This food was familiar and highly valued by all group members and was part of their daily mixed scatter feeds. To instil individually distinct, colour-driven food preferences in the two demonstrators (KEL, DW), the chow pellets were altered in size, taste and colour. KEL was trained to prefer blue and reject pink chow by repeated exposure to $1cm^3$ pieces of naturally tasting blue and bitter tasting pink chow. DW was trained in the opposite way, that is, by repeated exposure to $1cm^3$ pieces of naturally tasting pink and bitter tasting blue chow. When provided to the test subjects during foraging training the chow pellets were altered in size and colour only (i.e., $1cm^3$, pink or blue, see fig. S1). Colouration was obtained by soaking the chow in a mixture of water and blue or red artificial food colouring followed by air- or low temperature oven drying. The bitter taste was obtained by soaking the chow in a solution of water and a bitter additive (Bitrex® aqueous solution 2.5%, 0.35ml per 5cl of water: 175ppm). Pink and blue foods are rarely or never part of the group's regular diet.

Text S1.3 *Phase 1: Preference demonstration phase*

A demonstrator, either KEL or DW, was isolated and given the choice between two pellets of pink and two pellets of blue chow ten consecutive times, while the rest of the group observed from the opposite cage (cages 1 and 2, fig. 1). Food presentation lasted five seconds after a first choice had been made. Maintaining food presentation for 5 seconds allowed observers to witness the rejection of the second food item. An initial demonstration phase consisting of three

demonstration days per demonstrator was carried out early April 2015 and a reminder period of four demonstration days per demonstrator was carried out early June 2015. After this, refresher demonstrations took place at intervals of approximately 3-4 weeks. Subjects did not attend and observe all demonstrations. In a previous study, Shorland *et al*., (2019) found that subject exposure to food preference demonstrations was positively correlated to successful social learning of those food preferences, we therefore controlled for the exposure to demonstrations statistically. During winter months, playback experiments were not possible as the group as a whole was kept indoors, nonetheless a reminder period of two demonstration days per demonstrator was carried out in January 2016 and yet another, of four demonstration days per demonstrator in May 2016 before resuming playback experiments. After each playback trial, reminder demonstrations took place at intervals of approximately 4 weeks. Demonstrators KEL and DW chose their preferred colour first in 99.6% and 100% of trials respectively (N=240 for each demonstrator). All subjects attended to at least one preference demonstration during the initial demonstration phases in April and June 2015. On average, the 10 subjects attended to 30.2% of KEL's preference demonstrations (value is the mean percentage of feeding events observed).

Text S1.4 *Call recordings & stimuli*

A library of calls produced by KEL and DW for use as stimuli was built up in March, April and June 2015, calls from LNG were recorded in April and May of 2016. Food-associated call sequences produced by the demonstrator (KEL) and the control caller (LNG) were recorded during morning scatter feeds of chow (in its natural state) that were supplied to the group as a whole in their indoor enclosure following the first morning feed. Calls were recorded using a MARANTZ PMD660 solid-state recorder and a SENNHEISER MKH416T directional microphone. The caller was recorded from a distance ranging between 1m and 8m. In order to

identify calls produced by KEL or LNG, the experimenter (GS) focused on only one individual at a time and marked each call with a click that could later be visualized on a spectrogram. Call recordings were transferred onto an APPLE MacBook Pro and visualized in spectrogram format using the software Raven Pro 1.4 with a spectrogram window size of 512. Call sequences produced by KEL and LNG were identified, isolated, cleaned of click markings and background noise and saved as WAV audio files with a sampling rate of 44.1kHz with 16-bit accuracy before being imported into iTunes. Stimuli were played from iTunes on an APPLE MacBook Pro using a BOSE SoundLink Mini Bluetooth Speaker. Volume settings varied with each stimulus track and were defined by ear with help of the animal keeper by placing the speaker at a short distance and adjusting the volume setting so as to achieve the most realistic call amplitude for a bonobo at that distance. Call sequences were excluded when there was overlap with other calls or background noise. When possible, the stimuli were composed of the first four calls from a call sequence, a sequence being defined as a series of calls with call intervals shorter than one second (see fig. S5 and audio S1 for an example of a stimulus). When no such sequences were obtained from call recordings, four consecutive calls (max. call interval 36 sec.) were selected and call intervals were modified to replicate natural call intervals from existing call sequences. All stimuli were thus composed of four consecutive calls and lasted no longer than 4 seconds from the start of the 1st call to the end of the last call.

Text S1.5 *Criterion for subject participation*

The criterion for subject participation in the playback experiment was met when an individual, present in the 5m zone in front of the trapdoor during call playback, came to at least one of the two feeding troughs within the one-hour period following broadcast of the stimulus. During a one-hour period following stimulus playback, the experimenter (GS),

remained immobile, standing at equal distance from the two feeding troughs, facing cage 2 and gazing forward. Verbal observations were made for audio recording.
