# Supplementary material for: Bonobos assign meaning to food calls based on caller food preferences
Source: PLoS One. 2022 Jun 15;17(6):e0267574. doi: 10.1371/journal.pone.0267574 (PMC9200338; doi:10.1371/journal.pone.0267574)
Supplement: S1 File — Manual for the shorlandetal package for use with R. (PDF) [file pone.0267574.s020.pdf]

Manual for the shorlandetal package

Christof Neumann

2022-03-28

Prepare R

I suggest you use a recent version of R (>= 3.3) and ideally RStudio (> 1.0). Both are available freely (RStudio here, and R here).

In order to run the app contained in this package you need to have the following packages installed:

- shiny (version $\geq$ 1.0)
- lme4 (version $\geq$ 1.1.13)
- effects (version $\geq$ 3.0)
- rmarkdown

You can check whether you have them available with the following commands:

```
library("shiny")
library("lme4")
library("effects")
library("rmarkdown")
```

If either of these commands produces an *error*[1], you need to install the corresponding missing package(s), for example:

```
install.packages("shiny")
```

Repeat this until the loading step above does not produce *errors* anymore, i.e. you have all required packages available.

Install the shorlandetal package

Before you can use the app, you need to install the shorlandetal package. During the reviewing process, this is done with the supplemental .tar.gz file. Once the manuscript is accepted, we will upload the app to a public repository, which will make installation of it slightly easier.

To install the package use the following command (and adapt the file path to the location of the file according to your system).

On a Mac the command will probably look like this.

```
install.packages("~/Downloads/shorlandetal_1.0.1.tar.gz", repos = NULL, type = "source")
```

On Windows the command will probably look like this:

```
install.packages("c:/temp/shorlandetal_1.0.1.tar.gz", repos = NULL, type = "source")
```

[1] *warnings* can probably be ignored, if they inform you that some package has been built with some specific version of R

You can also install via RStudio's package management system by selecting "Installing from: Package Archive File" and then navigate to the `.tar.gz` file.

If this failed, the most likely reason is that your R version and/or some of the required packages are outdated. Please scan the error message you obtained to find which package is responsible and re-install it (`install.packages("packagename")`).

If this worked correctly, the following command will load the package and start the app.

```
library(shorlandetal)
bonobos()
```

While the app is running your R session will not be responsive. If you run the app from RStudio, just click the stop icon in the top right corner of your console to quit the app. Once that's done your R session will be responsive again.

Currently, the package only works if it is installed on a local drive, i.e. on your computer and not on some network drive. If you get an error message `Could not find app directory.` this means that you probably have R and/or its packages installed in a network location. You can verify this by running

```
.libPaths()
```

If this returns as first element something that contains something like '`\\\\home`' or similar it means that your R package library is primarily installed on a network drive. Currently, `shorlandetal` does not support running the app from such locations. Upon acceptance of the manuscript, we will make the app available online, which then won't require installation of this package in the first place.

Notes

This package version was created with R version 4.1.2 (2021-11-01), using `shiny` version 1.7.1, `lme4` version 1.1.28, `effects` version 4.2.1 and `rmarkdown` version 2.13. Originally it was created with `R` (v. 3.4.3), `shiny` (v. 1.1.0), `lme4` (v. 1.1.17), `effects` (v. 4.0.0) and `rmarkdown` (v. 1.9).

It has been tested on MacOS (v. 10.11.6) and Windows 7 (SP1).
